# Living With Assistive Robotics: Exploring the Everyday Use of Exoskeleton for Persons With Spinal Cord Injury

**DOI:** 10.3389/fmedt.2021.747632

**Published:** 2021-10-08

**Authors:** Roberto Lusardi, Stefano Tomelleri, Joseph Wherton

**Affiliations:** ^1^Department of Human and Social Sciences, Università di Bergamo, Bergamo, Italy; ^2^Nuffield Department of Primary Care Health Sciences, University of Oxford, Oxford, United Kingdom

**Keywords:** rehabilitation robotics, exoskeletons, technology-in-use, ReWalk, ethnography

## Abstract

**Background:** Recent advancements in sensor technology and artificial intelligence mechanisms have led to a rapid increase in research and development of robotic orthoses or “exoskeletons” to support people with mobility problems. The purpose of this case study was to provide insight into the lived reality of using the assistive robotic exoskeleton ReWalk.

**Method:** We used ethnographic techniques to explore the everyday experience and use of the assistive robotic device.

**Results:** We found that the appropriation and integration of the technology within the patient's everyday lives required a social and collaborative effort, which continued into use. The decisions to utilise the technology (or not) was closely tied to physical, social, cultural, environmental, and psychological factors. Consequently, there was much variation in patients' perception of the technology and opportunities for support. Four themes emerged:

(a) Meaning of mobility—physical mobility represents more than functional ability. Its present socio-cultural meaning is associated with an individual's self-identity and life priorities.

(b) Accomplishing body-technique—integration with the body requires a long process of skill acquisition and re-embodiment.

(c) Adaptation and adjustment in use—successful use of the technology was characterised by ongoing adjustment and adaptation of the technology and ways of using it.

(d) Human element—introduction and sustained use of the exoskeleton demand a social and collaborative effort across the user's professional and lay resources.

**Conclusions:** This study highlights that the development and implementation of the technology need to be grounded in a deep understanding of the day-to-day lives and experiences of the people that use them.

## Introduction

Recent advancements in sensor technology and artificial intelligence mechanisms have led to a rapid increase in research and development of robotic orthoses or exoskeletons to support people with mobility problems (1). These efforts have largely focused on technological progress to increasingly match the robot to the physical and motor characteristics of the human body (2–4). Whilst such developments are important, the psychosocial and cultural challenges of embedding such technology within patients' everyday lives have been overlooked, including physical, communication, learning, emotional, and motivational factors (5).

Rehabilitation robotics consists of robotic systems aimed at “(1) providing therapy for persons seeking to recover their physical, social, communication, or cognitive function, and/or (2) assisting persons who have a chronic disability to accomplish activities of daily living” [(5), p. 1686]. A third objective is outlined using artificial limb (prosthetics) and robotic exoskeletons in which the two primary objectives, therapy and assistance, can often coexist in the life of patients affected by conditions where functional recovery requires daily robotic support.

An exoskeleton is defined as an active mechanical device that is *worn* by an operator and fits closely to his or her body, and works in concert with the operator's movements. There has been a rapid increase in the number of robotic prostheses to support patients with lower-limb muscular weakness of disability. This is due to recent advancements in sensor technology and artificial intelligence mechanisms to match the robot to the physical and motor characteristics of the human body (2).

**Figure 1:**
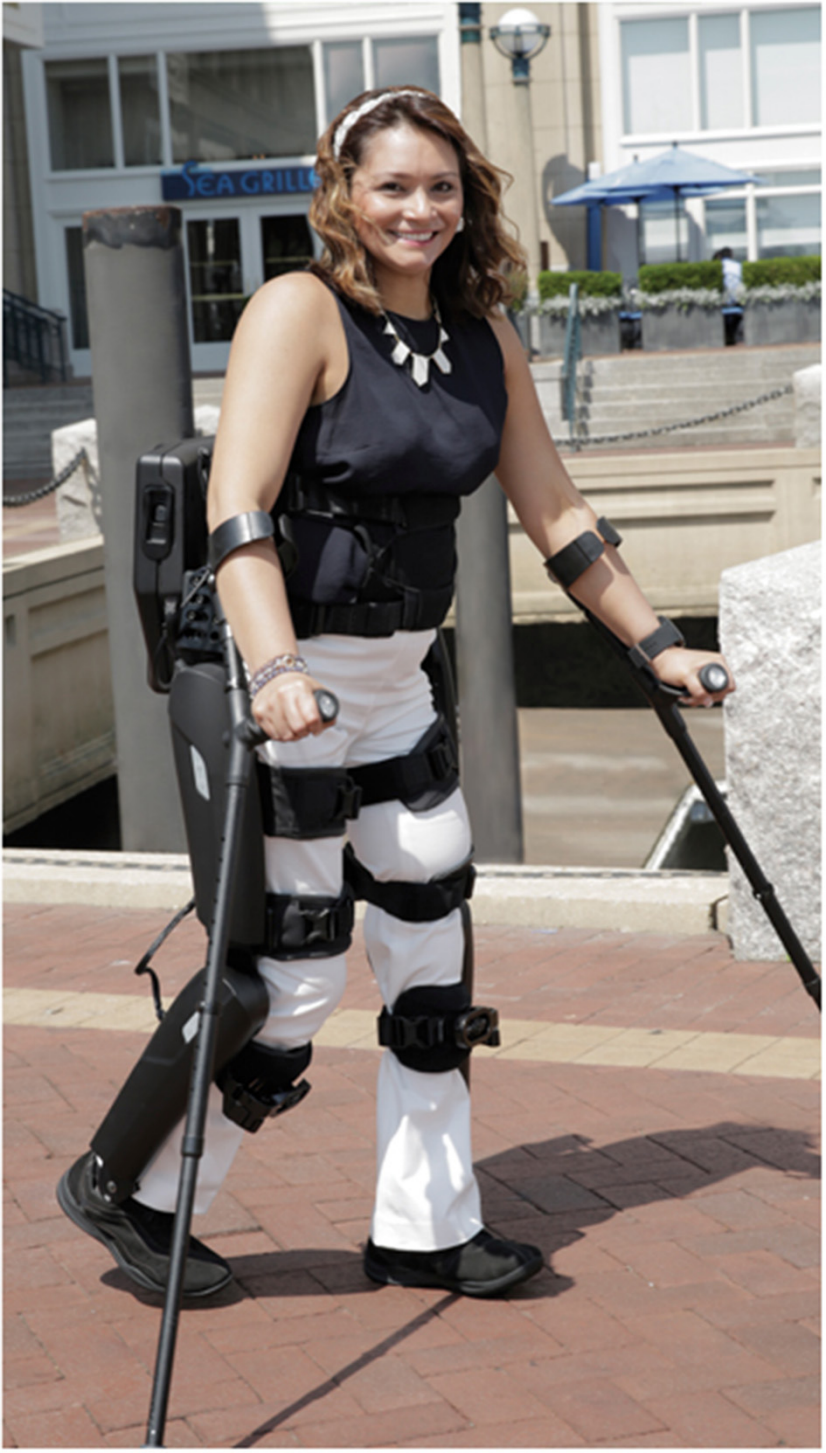
The ReWalk™ Exoskeleton. Source: https://rewalk.com/rewalk-personal-3/.

In this report, we focus on the ReWalk exoskeleton (https://rewalk.com/, retrieved 15/07/2021), designed to assist patients with complete spinal cord injury to walk independently, both indoors and outdoors (Figure 1). Its hip and knee joints are powered and controlled to follow a predefined trajectory. It includes a wrist-pad controller that allows the user to activate the movement, stand, sit and walk. It has a torso tilt sensor to trigger step-by-step transitions during walking.

Research into the appropriation and integration of robotic prostheses has largely focused on the technological progress and a segmented evaluation or “experimental validation” of the design against objective measures of performance, such as balance, gait, walking distance, velocity, stability, and duration (4, 6–8). But while technical performance is important, these advancements overlook the lived reality of disability and experience using robotic prostheses. It is important to consider how the technology relates to patients' everyday needs and priorities and how wider social and psychological issues influence the effective integration and use of the technology (9). While the repertoire of factors that intervene in human-machine interaction in the clinical setting [for a review, see (2)] is widely detailed, very little is known about what happens outside the walls of hospitals, laboratories, and research centres.

## Rational and Setting

The purpose of this study was to understand the lived reality of using an assistive robotic exoskeleton. To this end, we used ethnographic methods to explore the experience of implementing and using the technology from the perspectives of clinical staff and patients with spinal cord injury.

The research took place in the north of Italy, with staff and patients from a public medical centre for rehabilitative medicine devoted to recovering the best possible level of function of people with congenital or acquired disabilities. The hospital treats ~1,000 inpatients and 2,000 outpatients per year. The spinal injury rehabilitation team is led by a clinical neurologist and physiotherapist. The rehabilitation team consists of medical clinicians, nurses, occupational therapists, and physiotherapists specialising in recovery medicine.

The clinic provides rehabilitative programs for patients with complete and incomplete spinal injuries. This includes pharmaceutical and physical therapy to recover muscular and neural functioning and training with assisted living equipment. As part of their practise, the medical team introduce patients to and supported them with the ReWalk exoskeleton. ReWalk is a bionic suit with hip and knee joints powered and controlled to follow a predefined trajectory. With a wrist-pad controller, the user can activate the robotics system to perform *stand, sit* or *start walking*; with a torso tilt sensor, the user can trigger step to step transition during walking. The bionic suit detects shifts in the balance of the patient and moves the patient's leg in response. This procedure is more complicated than it appears. The movement of ReWalk is triggered and controlled by the position of the trunk, which is manipulated using the crutches. Hence there are three things that the patient must concentrate on to operate the device: (1) position of the trunk—the patient must perform the motion of leaning forwards and backwards to trigger the step movement; (2) the crutches—the patient must be aware of moving and positioning the crutches to move their trunk correctly; (3) feet position—the patient must track when a step has been completed to perform the next step.

Patients wishing to use ReWalk to assist them in everyday life must undergo an intensive training program before they are discharged with the technology. This training consists of 3–5 days a week, for 4–6 weeks. Each session lasts 90 min (with a short break in the middle). It takes ~10–15 min to put the technology on, with the assistance of the rehab team, and cheque that it is programmed correctly and securely fastened before proceeding.

In addition, at least one family member is trained alongside the patient so that they understand how the technology works and can provide on-hand assistance once the patient has been discharged. Patients are advised to not use the technology in the absence of someone who can assist them.

## Methods

To explore the lived reality of using ReWalk, we conducted ethnographic fieldwork at the rehabilitation clinic to capture the process of introducing the technology and conducting interviews with outpatients using the ReWalk at home.

Ethnographic fieldwork at the clinic included interviews and observations with staff and patients. The data collection focused on the introduction, training, and evaluation of the intervention. The research design and the data collection tools were submitted to the preliminary ethical assessment of the hospital governance, which approved them.

Interviews were conducted with two outpatients using the ReWalk exoskeleton at home. Both patients had paralysis below the waist. The interviews were conducted via Skype due to the geographical distance. Each participant was interviewed on two separate occasions, with each interview ~2 weeks apart. The interviews focused on how the technology helps support them inside and outside the home.

In addition, we developed and used cultural probes to support dialogue with patients during the interviews (10). The cultural probe method applies everyday artefacts and materials (e.g., cameras, diaries, wish lists) for participants to use in their own time and help narrate their lives to the researcher (11). It was previously found to be an effective way to explore the complex and often sensitive context of living with illness and disability (12). Following an initial interview, participants were introduced to the cultural probe materials, the “Home and Life Scrapbook” (Table 1) to complete in their own time for 1 week. The researchers went through each activity in turn, emphasising that they could choose which, if any, to complete. Table 1 shows the list of cultural probe activities.

**Table 1:** Summary of “home and life scrapbook” activities.

**Activity**	**Description**
Photos	Take any photos during the week.
Home map	Draw areas of the home and use different coloured pens to indicate different thoughts and feelings associated with them.
Places map	Draw maps of places outside the home that you go to (in green) or would like to go to but can't (in red).
Wishes	Three things they would like to improve or change about their lives.
Journey diary	Journeys or trips they choose to record (inside or outside), indicating what they liked about the journey and how it could have been better.
Memory box	Collection of existing photos that are important to them
Object box	Collection of objects that are important to them.

On the second interview (~2 weeks later), the researcher and participant reviewed and discussed the digital photos and scrapbook content together. Participants would either show the materials via the webcam or send them to the researchers electronically. The interview would focus on the materials collected by the participants.

The analysis was supported by existing literature on embodied movement (13), human geography (14), and sociotechnical systems (15). The latter is characterised by recursive relationship between technology and practise. Technologies are elements in complex, dynamic systems, in which use (or non-use) depends on human actions, interactions, and relationships as well as the material properties, affordances, and symbolic meanings of the technologies. Data for each case were drawn together using narrative synthesis to produce a case summary. The case narrative covered (a) the participant's social, cultural, and historical background; (b) their experience of their condition and the impact on mobility, (c) their use of the technology, (d) their perspective on what mattered about walking and mobility, (e) the specific exoskeleton technology that had been offered to support them; and (f) the problems that emerged, how these were resolved (or not) over time. The next section will summarise the key themes from the analysis.

## Findings and Discussion

### Meaning of Mobility

The patient case studies illustrated that physical mobility represents more than functional capacity. Rarely did patients use the technology to perform instrumental activities of daily living. Instead, the action of standing and walking presented socio-cultural meaning to the individual. Patients' uses of the technology were closely tied to their self-identity and life priorities, and hence unique to the individual. We observed that the patients using the technology appropriated and used the technology in very different ways, which were influenced by their dispositions, social and cultural contexts.

For example, one patient rarely used the technology in public, except for significant social events. He used ReWalk so that he could stand throughout his wedding ceremony. He also used the technology to stand when announcing to his family that his wife was pregnant.

Similarly, our other participant's use of the technology centred on exercise and improvement in physical strength. Often, he would head to the local athletics track with his family to walk. He was also exploring how the technology might be used to allow him to weight train. At present, the technology is not stable enough for him to lift heavyweight, and so he wanted to create a support that will allow him to engage in upper bodyweight training whilst standing.

These examples highlight the uniqueness of disability and how the appropriation of the technology needs to be grounded in an understanding of the individual's life. Hence, the design and provision of the technology should not focus on walking *per se* but instead centre on an understanding of *what matters* to the individual.

### Accomplishing Body-Technique

The use of the technology by the patient involves skill acquisition learned during their rehabilitation and training sessions. They must learn how to coordinate posture, movement, and balance while responding to visual and audio cues from the machine. Patients are not *relearning to walk* but instead are developing a completely new relationship with one's corporeality and self-image. Mauss (16) called it a *technique of the body*, a set of knowledge, awareness, and skills developed starting from the constant testing of our body in everyday activities and interactions with other people. Through time and experience, we develop some mastery of this technique. The use of robotic rehabilitative technology deeply questions this mastery and forces the person to reactivate the embodiment process.

Previous studies have described the process of re-embodiment in rehabilitative settings, such as the adoption of wheelchairs among spinal injured patients (17, 18). These studies have documented the process by which patients learn to act *through* the chair, in which their bodily awareness extends to include the frame of the device. The technological component enhances the expressions of the compromised body, expands its boundaries, redefines its potential and limits. Potentials and limits for the patient and the healthcare professionals themselves must be understood through the rehabilitation process and the “informal experiments” in daily life.

We have seen that our participants underwent this process of *re-embodiment* when learning to use ReWalk. This is considered necessary to use the technology effectively and safely, as one patient said during the interview:

“You find the right rhythm. You can find the right position. You pass through many difficulties and you also find solutions to those… The first week [of training] when you make the first decent steps, you feel euphoric. Then you tend to raise more your eyes, because when you start you look the ground for staring at where to put your feet”.

This process of re-embodiment with ReWalk centres on the cognitive load required to coordinate the system centres. Patients talked about “getting into the rhythm” of using the technology, in which they no longer need to make a conscious effort to perform the appropriate postures and actions to operate the technology.

It is important to acknowledge that this process takes time and effort (physical and mental) before the patient can master this new bodily style or technique. The ability to achieve this transition of the embodiment can vary greater among patients, depending on a range of factors, including the patient's level of coordination, concentration, and motivation.

It is important that technology developers are aware of how the mechanical workings of the device demand new body techniques and the impact that cognitive load has on the acceptance of the technology.

### Adaptation and Adjustment in Use

Previous studies have emphasised how people continually adapt and adapt to *technologies in-use*, including the use of assisted living technologies (19, 20). Our study has also revealed that effective integration of robotic exoskeletons demands ongoing adjustments and adaptations to meet the particular needs and capabilities of the user. We observed how patients continually adapted their physical and social environments in different ways to compensate for limitations in the technology and realise new possibilities for support. For example, one patient could not walk his dogs with the exoskeleton because he could not hold their leads (with the crutches in hand), and the machine is not stable enough for him to interact and play with the dogs (as they run around and jump up at him). However, he devised a pragmatic but effective solution to this. He decided to play with the dogs at a specific part of the garden where there was a wooden fence so that he could lean up against it for support. This then allowed him to interact with the dogs safely and comfortably.

These types of pragmatic and often subtle adaptations are important for achieving what matters to the individual. They highlight ways in which the technology could be improved to meet the particular needs of the patient in real contexts. Understanding how users feel constrained by technologies, or exploit opportunities to overcome them, provides valuable insights into how solutions can be improved. Therefore, the technology developers and suppliers need to work more closely with patients over time to gain insight into how patients confront and resolve the shortcomings of the technology and feed these back into the design cycle.

### Human Element

We found that the introduction and sustained use of the exoskeleton demands a social and collaborative effort across the user's professional and lay resources. For example, the introduction and training phase draws on the combined knowledge and expertise of staff, patients, and family members. Furthermore, the patient depends on the actions of others to assist in fitting, adjusting, and operating the technology once they are discharged with the technology. Patients are routinely advised never to use the technology in the absence of others so that someone is available to support them if they experience difficulties with the technology (e.g., lose balance), how one patient reported during the interview:

“Unfortunately I always need support by a person behind [me]…I must absolutely be assisted by another person. Everyone [practitioners] immediately gave me this small clause. I can walk alone, but there is always someone ready to run to help… However, they do not touch me. They let me go. I have the feeling of walking alone. The initial difficulty is not only to walk. The main difficulty is to dress, put the shoes. The main fear for a paraplegic person is to fall. You are on a basis that is not stable. So you have to find a balance”.

It is important, therefore, to understand assistive robotics as part of a socio-technical solution that needs to be developed and deployed in a way that is compatible with the social relations that make it work (9). We found that the need to involve others often led to a degree of selectivity about when and where it was appropriate to use the technology. For example, one patient never used his exoskeleton when socialising with friends because he did not want to impose them the responsibility to provide on-hand support if he had any difficulties. He felt that, in this social context, his wheelchair provided a greater level of independence.

Acknowledging the role of others in the integration and appropriation of robotics does not diminish the value of the technology as an assistive device. However, we must also be careful not to see the technology in isolation to make the person more independent from their social network. Instead, it is important to embrace the potential roles that others could play, alongside the technology, to enhance its potential role to help the patient participate and engage in meaningful activities.

## Concluding Remarks

Literature on the lived experience of robotic orthoses is scarce. To date, research and development has focused on the advancement and “validation” of the mechanical and software functionalities. However, our data has shown that technical progress alone will not address the challenges to the everyday use of robotic exoskeletons. Instead, we must shift focus toward the social and organisational processes that make the technology “work” for the individual patient.

This research highlights some key themes that need to be considered for the successful integration and appropriate robotic orthoses. Firstly, we have found that patients' mobility needs and wishes are unique (*meaning of mobility*). Robotic technology should not be seen primarily to enhance the functional status but as a tool to engage and participate in meaningful activities. Secondly, it is important to acknowledge the relationship between the body and the technology (*accomplishing body technique*). In particular, we should understand how the cognitive load required to operate the machine affects acceptance by the user. Thirdly, patients will continually adapt and adapt to the technology over time (*adaptation and adjustment in use*). Therefore, technicians and physicians need to find ways to continually track the use of the technology and support *ad-hoc* solutions to one-off problems. Finally, we must understand how the introduction and use of the technology align with the patient's formal and informal social support network (*human element*), in particular, how to support and sustain the social and collaborative efforts required for effective introduction and sustained use.

The lack of consideration for social and cultural issues within assistive and rehabilitative robotics research may be due to a dominant simplification of the man-machine hybridisation. Our preliminary findings illuminate a need to investigate the complex reality of using such technology, to inform the effective design and implementation of useful and useable solutions.

## Data Availability Statement

The raw data supporting the conclusions of this article will be made available by the authors, without undue reservation.

## Ethics Statement

The studies involving human participants were reviewed and approved by the research Ethics Committee at the local health care public institution. The patients/participants provided their written informed consent to participate in this study.

## Author Contributions

All authors listed have made a substantial, direct and intellectual contribution to the work, and approved it for publication.

## Funding

This study presented in this article was funded by the University of Bergamo through the grant Progetto ITALY^®^ - Azione 3: Grants for Visiting Professor and Scholar.

## Conflict of Interest

The authors declare that the research was conducted in the absence of any commercial or financial relationships that could be construed as a potential conflict of interest.

## Publisher's Note

All claims expressed in this article are solely those of the authors and do not necessarily represent those of their affiliated organizations, or those of the publisher, the editors and the reviewers. Any product that may be evaluated in this article, or claim that may be made by its manufacturer, is not guaranteed or endorsed by the publisher.
